# *Kdr*-based insecticide resistance in *Anopheles gambiae *s.s populations in

**DOI:** 10.1186/1756-0500-4-463

**Published:** 2011-10-28

**Authors:** Philippe Nwane, Josiane Etang, Mouhamadou Chouaїbou, Jean Claude Toto, Rémy Mimpfoundi, Frédéric Simard

**Affiliations:** 1 Organisation de Coordination pour la lutte contre les Endémies en Afrique Centrale, Yaoundé, Cameroun; 2 Université de Yaoundé I, Yaoundé, Cameroun; 3 Faculty of Medicine and Pharmaceutical Sciences, University of Douala, Cameroun; 4 Institut de Recherche pour le Développement (IRD), UR016, Bobo-Dioulasso, Burkina Faso

## Abstract

**Background:**

The spread of insecticide resistance in the malaria mosquito, *Anopheles gambiae *is a serious threat for current vector control strategies which rely on the use of insecticides. Two mutations at position 1014 of the S_6 _transmembrane segment of domain II in the voltage gated sodium channel, known as *kdr *(*knockdown resistance*) mutations leading to a change of a Leucine to a Phenylalanine (L1014F) or to a Serine (L1014S) confer resistance to DDT and pyrethroid insecticides in the insect. This paper presents the current distribution of the *kdr *alleles in wild *Anopheles gambiae *populations in Cameroon.

**Results:**

A total of 1,405 anopheline mosquitoes were collected from 21 localities throughout Cameroon and identified as *An. gambiae *(N = 1,248; 88.8%), *An. arabiensis *(N = 120; 8.5%) and *An. melas *(N = 37; 2.6%). Both *kdr *alleles 1014F and 1014S were identified in the M and S molecular forms of *An. gambiae *s.s. The frequency of the 1014F allele ranged from 1.7 to 18% in the M-form, and from 2 to 90% in the S-form. The 1014S allele ranged from 3-15% in the S-form and in the M-form its value was below 3%. Some specimens were found to carry both resistant *kdr *alleles.

**Conclusion:**

This study provides an updated distribution map of the *kdr *alleles in wild *An. gambiae *populations in Cameroon. The co-occurrence of both alleles in malaria mosquito vectors in diverse ecological zones of the country may be critical for the planning and implementation of malaria vector control interventions based on IRS and ITNs, as currently ongoing in Cameroon.

## Background

Insecticide resistance is a major concern in all insect groups that are involved in crop destruction or in disease transmission. Four different types of mechanisms including behavioural avoidance, reduction of cuticle penetration, metabolic detoxification and reduced target-site sensitivity lead to insecticide resistance in many arthropod groups [1]. So far, metabolic detoxification and target site insensitivity have been demonstrated to play major roles in conferring resistance to insecticides in some arthropods [2]. While metabolic resistance is due to changes in the arthropod enzyme activity resulting in the detoxification or sequestration of the insecticide, target site insensitivity is due to mutations preventing the binding of the insecticide to its target [3].

The target site of DDT and pyrethroid insecticides is the voltage-gated sodium channel. Different point mutations identified in the S_6 _transmembrane segment of domain II of this para-type sodium channel gene cause a change in affinity between insecticide and its binding site. This induces a phenotype termed knockdown resistance (*kdr*) in a wide range of insects [4-6]. Different amino-acid substitutions occurring at variable positions on the voltage-gated sodium channel have been reported in several studies. In most cases, the substitution of a Leucine residue to a Phenylalanine was commonly noted. At position 1014 the substitution of a Leucine residue to a Phenylalanine (L1014F) is observed in a range of arthropod species, including *Musca domestica *[7], *Myzus persicae *[8], *Plutella xylostella *[9], and the mosquito *Anopheles gambiae *[5], the major vector of human malaria in Africa. Another widespread mutation changes the Leucine at position 1014 to a Serine (L1014S) in wild populations of *An. gambiae *[6]. In *An. gambiae*, the L1014F mutation is widely distributed in West and Central Africa, whereas the L1014S mutation has a much more restricted geographic range in Eastern Africa [10]. The spread of these mutations in wild populations of *An. gambiae *threatens the effectiveness of malaria vector control strategies based on the use of chemical insecticides, and prompts for surveillance and monitoring [11].

Today, pyrethroid insecticides are most recommended for use in public health because of their high effectiveness and strong excito-repellent effect on insects, as well as low mammalian toxicity [12,13]. These insecticides make up around 40% of chemical insecticides used globally each year for indoor residual spraying of houses against malaria mosquitoes, and 100% of the WHO-recommended insecticides for the treatment of mosquito nets are pyrethroids [14]. Vector control is a key strategy in reducing malaria transmission and prevalence in endemic countries [15,16]. This strategy is chiefly based on the use of chemical insecticides for indoor residual spraying (IRS) and impregnation of bed nets for killing adult mosquitoes [17-19]. Pyrethroid-impregnated nets are therefore being massively scaled-up in Africa, but there is serious concern about the likely evolution of widespread pyrethroid resistance among *Anopheles gambiae *mosquito populations.

In Cameroon, insecticide treated nets (ITNs) are used for malaria vector control since the year 2000, although implementation varies depending on local capacity [20]. The level and spread of resistance to DDT and pyrethroids (deltamethrin, permethrin, lamda-cyalothrin) has been reported in several malaria vector populations mainly in *An. gambiae *s.s and *An. arabiensis *[21-23]. Moreover, it was demonstrated that enzyme systems such as esterases, gluthatione S-transferases and cytochrome P_450 _monooxygenases are implicated in the resistance of these mosquito populations [24-26], and both *kdr *mutations were reported [27]. However, little is known about the geographic distribution and frequency of both *kdr *mutations throughout the country.

The current report provides a detailed update of the occurrence, frequency and geographic distribution of both L1014F and L1014S *kdr *mutations within and among *An. gambiae *populations from throughout Cameroon.

## Methods

### Study sites

Mosquitoes were sampled in 21 locations (Table 1) spanning the whole of Cameroon, and spread across its four main geographic areas:

**Table 1:** Molecular identification of members of the *Anopheles gambiae *complex collected in Cameroon.

Geogra- phic area	Locality	Geographic coordinates	Sampling period	Sampling method	Climatic and ecological domains	*An. arabiensis*	*An. gambiae *s.s		*An. melas*
								
							M-form	S-form	
Forest	Ngousso	03°53'44"N-11°3318"E	May 2006	LC	Equatorial forest, urban	-	57	6	-
	Nkolondom	03°56'52"N-11°3018"E	Dec 2005	LC	Equatorial forest, market gardening area	-	-	64	-
	Dabadi	05°36'10"N-13°37'50"E	May 2006	LC	Equatorial/Tropical, urban	-	-	72	-
	Italie	05°36'07"N-13°44'22"E	May2006	LC	Equatorial/Tropical, urban	-	-	75	-
	Nkolbikon	05°36'06"N-13°40'30"E	May 2006	LC	Equatorial/Tropical, urban	-	22	55	-

Coastal	Ipono	02°22'29"N-09°52'28"E	Dec. 2005	LT+CA	Equatorial, humid forest, rural	-	22	14	37
	Campo	02°22'30"N-09°49'33"E	Dec. 2005	LC+CA	Coastal equatorial, humid forest, rural	-	37	39	-
	Kribi	02°56'33"N-09°54'26"E	Dec. 2005	LC	Coastal equatorial, humid forest, urban	-	62	11	-
	Bonamikengué	03°48'18"N-10°08'08"E	Oct. 2005	LC	Coastal forest, urban	-	64	4	-
	Bonanloka	04°01'43"N-09°43'54"E	May 2005	LC	Coastal, equatorial, urban	-	38	24	-
	Bonanjo	04°02'22"N-09°41'13"E	Oct. 2005	LC	Coastal equatorial, urban	-	61	2	-
	Bonassama	04°04'26"N-09°41'06"E	Oct. 2005	LC	Coastal equatorial, urban	-	74	-	-
	Loum	04°42'13"N-09°44'03"E	Oct. 2005	LC	Equatorial forest, suburban	-	77	-	-
	Tiko	04°05'22"N-09°21'09"E	Nov. 2005	LC	Equatorial forest, urban,	-	47	18	-
	Idenau	04°13'23"N-08°58'13"E	Nov. 2005	LC	Coastal equatorial, suburban	-	18	-	-

Highland	Mangoum	05°28'35"N-10°35'18"E	Oct. 2005	LC	Tropical, grassland mountains, market gardening area	-	-	76	-
	Makoutchietoum	05°36'37"N-10°36'24"E	Oct. 2005	LC	Tropical, grassland mountains, market gardening area	-	-	77	-
	Magba	05°58'10"N-11°13'38"E	Oct. 2005	LC	Tropical, transition forest/savanna, rural	1	-	62	-
Northern savanna	Tibati	06°28'12"N-12°37'20"E	May 2007	LC+LT	Tropical, humid savanna, suburban	14	-	50	-
	Ngaoundéré	07°19'04"N-13°35'38"E	Oct. 2006	LC	Tropical, humid savanna, urban	45	-	16	-
	Pitoa	09°23'31"N-13°30'09"E	Oct. 2006	LC	Tropical, dry savanna, suburban, cotton area	60	-	4	-

LC: larval collection, LT: Light trap; CA: capture with aspirators

i) the forest area located in the southern part of the country which extends from latitude 2° to 6° North and experiences typical Equatorial Guinean climate with average yearly rainfall between 1,500-2,000 mm spread out over 4 seasons: 2 dry seasons (December-February and July-August) and 2 rainy seasons (March-June and September-November). Mean annual temperature is 25°C [28]. Five localities were sampled in this area (Table 1).

ii) the coastal area situated alongside the Atlantic ocean, exposed to equatorial climate characterized by a long rainy season (March-November) with high annual rainfall between 2,000-10,000 mm and average annual temperature at 26°C [29]. A total of ten localities were visited in this area (Table 1).

iii) the western highlands located in the South-Western region of Cameroon. The area is characterized by one dry season between November and February and one rainy season between March and October with a mean annual rainfall of 1,800-2,500 mm and average yearly temperature below 22°C [28]. In this part of the country, mosquito collections were carried out in three localities (Table 1).

iv) the northern savannas exposed to tropical climate, subdivided into the humid tropical and Sahelian climate domains [28,29]. The humid tropical domain extends from about latitude 6° to 10° North and is characterized by 2 seasons: one dry season from November to May and one rainy season from June to October with an average yearly rainfall between 700 and 1,000 mm, and mean annual temperature around 26°C. The Sahelian climate domain encompasses the northernmost areas of the country, North of the Benue basin. The region receives annual rainfalls below 900 mm, and experiences a long dry season of more than 7 months (October-May) with annual temperature around 28°C [28]. Mosquitoes were collected in three localities (Table 1).

### Mosquito collections and species identification

Mosquitoes were collected between May 2005 and May 2007 according 3 sampling methods [30]:

i) the dipping method (LC in Table 1), used to collect anophelines larvae and pupae from breeding sites using tanks, ladles, sieves and pipettes. In each study site, collections were performed in 10-15 breeding sites with 10-20 larvae collected per breeding site and reared locally until adult emergence;

ii) the indoor resting collection method, used to collect adult mosquitoes with mouth operated aspirators in human dwellings (CA in Table 1);

iii) the light trap method, used for the collection of anthropophagic adult mosquitoes with miniature light traps operated in dwellings during the night (LT in Table 1).

Adult mosquitoes were morphologically identified in the field using reference keys [31,32]. They were stored individually in labelled tubes with a desiccant and kept in storage boxes at -20°C in the laboratory for further analyses.

### Molecular identification and *kdr *genotyping

DNA was extracted from each mosquito specimen using the method of Collins and colleagues [33] and individual mosquitoes were identified down to their species and molecular form using PCR-RFLP [34]. This method allows simultaneous identification of the M and S molecular forms within *An. gambiae *s.s, as well as the other species of the *An. gambiae *complex. *Kdr *alleles were genotyped using hot oligonucleotide ligation assay (HOLA) as described by Lynd and colleagues [35].

### Statistical analysis

Proportions of molecular forms and *kdr *allele frequencies with their respective confidence intervals were determined using bootstrap statistical inference. The method is based on building a sampling distribution by re-sampling from field collected data. Data processing was performed using Excel and R softwares (R Development Core Team, 2005). The distribution of genotypes at the *kdr *locus was tested for conformity to Hardy-Weinberg equilibrium within each molecular form and collection site, using exact tests available in GENEPOP 3.3 software [36].

## Results

A total of 1,405 anopheline mosquitoes from the *An. gambiae *complex were collected in 21 sampling sites with at least 60 specimens per site, except in Idenau (coastal area) where only 18 individuals were collected (Table 1).

### Species and molecular form distribution

Three anopheline species were identified among the specimens collected in the 21 prospected sites: *An. melas, An. arabiensis *and *An. gambiae s.s*.. *Anopheles melas *was collected at the adult stage in Ipono where it represented 50% of the total number of mosquitoes collected in this locality situated in the mangrove area of coastal Cameroon. *Anopheles arabiensis *was collected in the western highlands and in the northern savannas areas, at increasing frequencies when moving northwards (Table 1). *Anopheles gambiae *s.s was sampled in all sites: it was the only species of the complex surveyed in the southernmost sites, and decreased in frequency when moving northwards (Table 1). Both M- and S-form mosquitoes were found among the samples and occurred together in 9/21 localities (Table 1). The M-form was widespread and predominant in the coastal area characterized by abundant rainfalls and maximum relative humidity, as well as in large urban centres in the forest area. No *An. gambiae *M-form was found in the highlands area, as well as in the northern savannas. The S molecular form was found in 18/21 sites, being predominant in the rural areas and suburban zones (Table 1). In the highlands and northern savannas areas, *An. gambiae *s.s. samples were essentially made up with the S molecular form. Proportions of this molecular form were < 40% in the coastal area, except in Campo where the proportions of the 2 forms were nearly equal. No M/S hybrid was found in our samples even in sites where M and S were sympatric.

### Distribution of the *kdr *alleles

**Figure 1:**
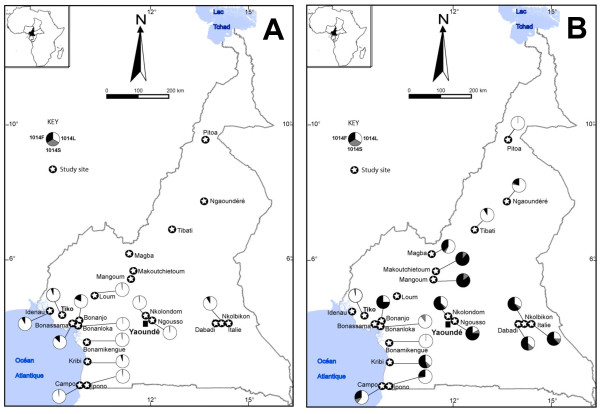
**Distribution of 1014L, 1014F and 1014S kdr alleles in Anopheles gambiae M form (A) and S form (B) populations**.

All three *kdr *alleles (1014L, 1014F and 1014S) were detected in both molecular forms of *An. gambiae *s.s. (Table 2), although at markedly different frequencies and with strong geographical variation within form. In the M molecular form, the 1014F allele was detected in 7/12 samples, at a frequency always below 20% (Table 2 Figure 1A). The highest frequencies were observed in the coastal area, especially in Bonanjo and Bonassama which are two central districts of Douala, the biggest harbour of Cameroon. In Nkolbikon, in the easternmost part of the forest area of South Cameroon where the 1014F allele occurs at c.a. 7% in the M-form population, the 1014S allele was observed in one M-form specimen, at the heterozygous state.

**Table 2:** Frequency of *kdr *alleles in *Anopheles gambiae *s

*An. gambiae *s.s	Geographic area	Locality	N	Allelic frequencies (%)			F_IS_	p(HW)
						
				f (1014L)[95%CI]	f (1014F)[95%CI]	f (1014S)[95%CI]		
M-form	Forest area	Ngousso	57	98.2 [95.5-100]	1.7 [0-4.5]	0	- 0.009	0.991
		Nkolbikon	22	90.9 [79.2-100]	6.8 [0-18.2]	2.3 [0-7.5]	-	-
	
	Coastal area	Ipono	22	100	0	0	-	-
		Campo	37	100	0	0	-	-
		Kribi	62	95.2 [91.2-98.4]	4.8 [1.5-8.8]	0	- 0.043	0.877
		Bonamikengué	64	100	0	0	-	-
		Bonanloka	38	100	0	0	-	-
		Bonanjo	61	81.9 [73.8-89.5]	18.0 [10.5-26.2]	0	**+ 0.342**	**0.027**
		Bonassama	74	87.2 [80.4-93.2]	12.8 [6.8-19.6]	0	**+0.462**	**0.011**
		Loum	77	100	0	0	-	-
		Tiko	47	94.7 [90-98.9]	5.3 [1.1-10]	0	- 0.045	0.911
		Idénau	18	91.7 [83.3-100]	8.3 [0-16.7]	0	-	-

S-form	Forest area	Ngousso	6	16.7 [0-50]	75.0 [35.7-100]	8.3 [0-25]	-	-
		Nkolondom	64	35.9 [25.8-46.1]	60.2 [50-70.3]	3.9 [0.8-8.6]	**+0.452**	**0.005**
		Dabadi	72	39.6 [31.9-47.9]	46.5 [38.2-54.2]	13.9 [8.3-19.4]	- 0.001	0.583
		Italie	75	25.3 [19.3-32.0]	62.7 [55.33-70]	12.0 [7.3-17.3]	- 0.103	0.172
		Nkolbikon	55	32.7 [24.1-41.2]	60.9 [51.7-70.2]	6.4 [2.5-11.2]	+ 0.019	0.538
	
	Coastal area	Ipono	14	71.4 [54.2-87.5]	25.0 [11.5-38.2]	3.6 [0-11.5]	-	-
		Campo	39	60.2 [47.1-73.3]	29.5 [17.9-41.9]	10.3 [3.6-18.2]	**+ 0.346**	**0.005**
		Kribi	11	36.4 [13.6-60]	54.5 [33.3-75]	9.1 [0-22.2]	-	-
		Bonamikengué	4	100	0	0	-	-
		Bonanloka	24	89.6 [78.6-98.1]	10.4 [1.9-21.4]	0	-	-
		Bonanjo	2	25.0 [0-50]	75.0 [0-100]	0	-	-
		Tiko	18	97.2 [90.6-100]	2.8 [0-9.4]	0	-	-
	
	Highland area	Mangoum	76	0.6 [0-1.9]	84.9 [79.6-90.1]	14.5 [9.2-19.7]	+0.001	0.577
		Makoutchietoum	77	0.6 [0-1.9]	88.3 [83.8-92.9]	11.0 [6.5-15.6]	-0.1350	0.250
		Magba	62	57.3 [47.6-66.9]	37.9 [29.8-46.8]	4.8 [1.6-8.9]	+0.088	0.161
	
	Northern savannah area	Tibati	50	91.0 [83-97]	9.0 [3-17]	0	**+0.631**	**0.005**
		Ngaoundéré	16	78.1 [56.2-93.7]	18.7 [3.1-37.5]	3.1 [0-9.4]	-	-
		Pitoa	4	100	0	0	-	-

f(): frequency of the allele (in %); [95%CI]: 95% confidence interval; N: number of mosquitoes; p(HW): probability of the exact test for goodness of fit to Hardy-Weinberg equilibrium; **in bold**: Significant value (p(HW)<0.05, single test level); Fis is calculated according to Weir and Cockerham, 1984. Positive Fis indicates a deficit of heterozygotes and negative Fis indicates an excess of heterozygotes; -: not determined because no polymorphism observed and/or N < 30.

Elsewhere (e.g in 5/12 localities), all M-form specimens carried the susceptible 1014L allele at the homozygous state. Hardy-Weinberg proportions were generally respected, except in two cases in M-form populations from the coastal area (Table 2). These significant departures were associated with a deficit in heterozygotes. In the S molecular form, the 1014F allele was observed in all sites, except in 2 locations where sample sizes were very low (N = 4, Table 2). The frequency of this allele ranged from ≈3% in Tiko (coastal area) to 88% in Makoutchietoum (highland area). Globally, the highest frequencies of this allele were recorded in the highlands and forest areas, and were lowest in the northern savannas (Table 2 Figure 1B). The 1014S allele was detected in 12 out of 18 S-form samples, at a frequency ranging from 3 to 15%. The allele was spread throughout all ecological zones of Cameroon, reaching its highest frequencies in S-form populations from the forest and highlands areas. Significant (p<0.05, single test level) departures from Hardy-Weinberg proportions were observed in 3 out of 10 populations tested (Nkolondom, Campo and Tibati, Table 2). These departures were associated with deficits in heterozygotes. All *An. arabiensis *(N = 120) and *An. melas *(N = 37) specimens tested were homozygous for the susceptible 1014L allele at the *kdr *locus.

## Discussion

The distribution of species within the *An. gambiae *complex observed in this study is in agreement with the known biology of these taxa [32,37]. As for the M and S molecular forms of *An. gambiae s.s*, their distribution and ecological requirements agree with previous studies carried out in Cameroon [38-40]. The presence of *An. arabiensis *was almost exclusive in the northern savanna area characterized by a drier climate and mean annual rainfall below 1,000 mm, in agreement with the ecological features described in previous studies. *Anopheles melas *was identified in Ipono, a rural locality situated in the mouth of the Ntem river in the coastal area. The locality is surrounded by mangrove swamps, which are typical breeding sites for this species. *Anopheles melas *represented c.a. 50% of the total mosquitoes collected at the adult stage in this locality, confirming its anthropophilic and endophilic behavior [38,41,42].

*An. gambiae *s.s. was the most frequent species of the complex collected during this study. This species exhibits two distinctive molecular forms termed M and S characterized by fixed nucleotide difference in the intergenic spacer of the ribosomal DNA [43,44]. Genetic differentiation between these molecular forms is high only in two or three tiny genomic areas named the speciation islands (representating 1% of the total genome), with low or no differentiation found across most of the genome [45-47]. It is likely that the M and S molecular forms are distinct species [48,49], and there are distinct differences in the assortment of insecticide resistance genotypes and phenotypes between them. In this study, the two molecular forms of this species were identified in the samples, occurring at various relative frequencies from one site to another. The absence of MS hybrids reinforces previous findings for strong genetic isolation of the M and S forms in Cameroon [39,50,51]. Globally, the M molecular form was widespread in the highly urbanized coastal area and in around major urban centres in the forest area. Its distribution was restricted to the southernmost localities, and it was not found above latitude 5°N. The S form was more widespread and was found in all geographical areas sampled, albeit at a lower frequency than the M form in urbanized areas in the South. It was highly predominant in the central part of the country including western highlands and occurred together with *An. arabiensis *in northern savannas. This distribution pattern is consistent with previous reports [22,38,39,52]. Physical environmental factors such as temperature, water vapour pressure, evapotranspiration, sunlight exposure, annual rainfall and land cover have been shown to influence the distribution of the M and S forms of *An. gambiae *at the geographical scale of the country in Cameroon [39]. Other biotic and abiotic factors need to be involved to explain the heterogeneous distribution observed at a more local geographical scale. In this regard, it is important to stress that these vector populations typically show temporal variations in their relative abundance [23]. This might lead to mosaic patterns of species occurrences along a geographical transect, such as observed in the present study in areas where the habitat is globally equally favourable to both species/molecular forms. Moreover, biotic interactions occurring at the larval and/or adult stages such as competition, predation and parasitism might further determine the population's structure and impact on species balance locally, as recently evidenced from studies conducted on *An. gambiae *populations of the M and S form in Burkina Faso [53,54]. Finally, anthropogenic factors, such as chemical insecticide usage in public health and agriculture may be a key factor in selecting one or the other of the molecular form, according to the resistance mechanism(s) it is armed with.

The coexistence of both 1014F and 1014S *kdr *alleles was evidenced in the M molecular form as well as in the S form in this study, although both alleles occurred at a much higher frequency in S-form than in M-form populations. Some authors have suggested that there is a link between the 1014F and 1014S *kdr *alleles and resistance phenotypes to DDT and pyrethroid insecticides in field populations of *Anopheles gambiae *s.s [5,55,56]. However, questions over the reliability of inferring resistance phenotype based solely on the diagnosis of *kdr *genotype have been raised, because correlations between phenotype and *kdr *genotype are obscure in some instances [57]. Other authors argue that the *kdr *alleles cannot alone confer resistance to DDT and pyrethroid insecticides in the absence of hypothetical, thus far unidentified co-factors [57,58]. Alone or in combination with the high activity level of detoxification enzyme systems (monooxygenases, glutathione-S-transferases and non-specific esterases) reported in some mosquito populations of our study area [24-26], the 1014F and 1014S *kdr *alleles identified in most of our collection sites could have an impact on the high levels of *An. gambiae *s.l resistance to these insecticides reported throughout Cameroon [59]. On the whole results presented in this study shows a rise in frequency of resistant *kdr *alleles (e.g., 1014F and 1014S) in both molecular forms compared to previous studies related to the frequency of these alleles in the country [22,23,27].However, these frequencies, particularly that of the 1014F recorded in the M molecular form are lower compared to that obtained in a recent survey carried out in Cameroon where the frequency of this allele was 68% in Douala and 44% in Yaounde [60]. These alleles were not detected in *An*. *arabiensis *nor *An. melas *specimens. None of them has not yet been identified in *An. melas *whereas several studies reported their presence in *An. arabiensis *in Kenya [61], Sudan [62,63] and Cameroon [22]. The absence of these alleles in the present samples suggests their recent introduction in *An. arabiensis *in Cameroon where they seem to occur at a very low frequency. Within *An. gambiae *s.s, both 1014F and 1014S alleles were previously reported in Cameroon [27,64]. In neighbouring countries, at least one of these alleles has been found, *e.g*. in Equatorial Guinea [65], Gabon [66], Central Africa Republic [67], Chad [68] and Nigeria [69] emphasizing the spread of the *kdr *mutations in Central Africa [10]. However, again, referring to results based on cross sectional studies calls for caution and care must be taken in predicting absence of these alleles in a given zone/area of the country.

The uneven distribution of *kdr *alleles between molecular forms and species of the *An. gambiae *complex probably reflects different molecular evolution dynamics within species and forms and different levels of exposure to insecticide-driven selection pressure [10,70-72]. A significant heterozygote deficit was noted in some M and S form populations when *kdr *genotypic frequencies were compared to Hardy-Weinberg proportions. This heterozygote deficit was observed in sites where chemical insecticides particularly pyrethroids have been reported to be commonly used for agriculture, wood/forest exploitation and public health purposes [23,72]. Although the resistance genotype was not determined in this study, we think the selection pressure from agricultural use of both DDT and pyrethroids, as well as to DDT-based vector control campaigns undertaken in the 1950s may confer a selective advantage to resistant homozygote individuals because *kdr *is a recessive trait [5].

The selection of the *kdr *alleles has been evidenced with the use of ITNs [73,74]. ITNs or LLINs are distributed on a large scale in Cameroon since ≈10 years by the National Program of Malaria Control. This may constitute an additional source for the selection pressure of *kdr *alleles in several *Anopheles gambiae *mosquito vector populations in Cameroon. Here, the highest frequencies of *kdr *alleles were recorded in urban areas (*e.g.*, Ngousso, Dabadi, Nkolbikon and Italie) and agricultural settings (*e.g*., Nkolondom, Campo and Makoutchietoum) where large amounts of chemical insecticides are commonly used for diseases vector control, personal protection against nuisances and crop/wood protection [23]. The 1014F and 1014S *kdr *alleles were initially identified in *Anopheles gambiae *mosquitoes from West [5] and East [6]. Africa respectively. Their co-occurrence and rise in frequency in mosquito vector populations in Cameroon testifies of the ongoing geographical spread of both alleles invading wild *Anopheles gambiae *populations throughout Africa.

## Conclusions

In West Africa, ITNs were reported to provide personal protection even against *kdr-*based resistant *An. gambiae *populations [75-77], but recent studies suggested reduced efficacy of ITNs and IRS in areas with high frequencies of 1014F *kdr *allele [78]. High frequencies of *kdr *alleles in malaria mosquito populations in Cameroon prompts the need for close monitoring of vector susceptibility levels to insecticides and tracing of resistance mechanisms in order to devise adapted vector control measures and prevent failure in areas where these methods are implemented.

## Competing interests

The authors declare that they have no competing interests.

## Authors' contributions

JE and FS conceived the study. JE, FS, RM, and PN designed the study protocol; JE, PN, MC and JCT performed field work and bioassays; PN performed molecular analyses, interpreted the data and drafted the manuscript which was critically revised by JE, RM, MC and FS. All the authors read and approved the final manuscript.
